# Interaction between the impact of the Coronavirus disease 2019 pandemic and demographic characteristics on sexual/erectile dysfunction in Latin America: cross-sectional study

**DOI:** 10.1590/S1677-5538.IBJU.2021.0764

**Published:** 2022-03-14

**Authors:** Constanza Alvear Pérez, Luciana de Barros Cavalcanti Michelutti, Maria Volpato Palharini, Luisa Pasqualotto Teixeira, Valeria Regina Silva, Lucas Emmanuel Pedro de Paiva Teixeira, Silvia Lanziotti Azevedo da Silva, Simone Botelho

**Affiliations:** 1 Universidade Federal de Alfenas Instituto de Ciências Motoras Ciências da Reabilitação Alfenas MG Brasil Programa de Pós-Graduação em Ciências da Reabilitação, Instituto de Ciências Motoras da Universidade Federal de Alfenas - UNIFAL-MG, Alfenas, MG, Brasil; 2 Universidade Federal de Alfenas Faculdade de Medicina Alfenas MG Brasil Faculdade de Medicina da Universidade Federal de Alfenas - UNIFAL-MG, Alfenas, MG, Brasil; 3 Universidade Estadual de Campinas Faculdade de Ciências Médicas Pós-Graduação em Ciências Cirúrgicas Campinas SP Brasil Programa de Pós-Graduação em Ciências Cirúrgicas, Faculdade de Ciências Médicas da Universidade Estadual de Campinas – UNICAMP, Campinas, SP, Brasil; 4 Universidade José do Rosário Vellano Alfenas Brasil Universidade José do Rosário Vellano - UNIFENAS, - Alfenas, MGm Brasil; 5 Universidade Federal de Juiz de Fora Federal de Juiz de Fora Juiz de Fora MG Brasil Departamento de Saúde Coletiva, Faculdade de Medicina, Universidade Federal de Juiz de Fora – UFJF, Juiz de Fora, MG, Brasil

**Keywords:** Sexual Dysfunction, Physiological, Coronavirus Infections, Stress Disorders, Traumatic

## Abstract

**Aim:**

Our objective was to investigate whether there is an interaction between the COVID-19 pandemic, demographic characteristics and erectile/sexual (E/S) function in individuals from Latin America.

**Materials and Methods:**

Cross-sectional study which included Latin American individuals over 18 years old, recruited through social media and interviewed between July and August 2020 by online surveys (Google Forms) in Portuguese and Spanish languages. The E/S function was evaluated through the following questionnaires: Simplified International Index of Erectile Function (IIEF-5) and Female Sexual Function Index (FSFI); while post-traumatic stress disorder (PTSD) triggered by the COVID-19 pandemic was assessed through the Impact of Event Scale Revised (IES-R). The data was analyzed by T Student, bivariate and multivariate logistic regression, with significance determined by the Wald test (p<0.05), using the R software v4.0.

**Results:**

Out of the 2016 individuals that responded to the survey, 1986 were included and 743 of them presented E/S dysfunction. PTSD occurrence was greater among people with E/S dysfunction when compared to those without E/S dysfunction, in the total score (males: IES-R=26.54[±19.17] and females: IES-R=35.92[±19.25]) and also in the three domains. It was found that those who do not live with a partner were 74% more likely to have E/S dysfunction, but living with a partner during the pandemic had a greater impact on E/S function.

**Conclusion:**

A negative interaction between the impact of the COVID-19 pandemic and erectile/sexual function of the Latin American population was observed, with greater implications among the individuals who live with their partners.

## INTRODUCTION

In March 2020, the Coronavirus disease 2019 (COVID-19) pandemic was decreed, starting in Wuhan, China and rapidly affected the whole world, due to its propagation by aerosols and/or droplets (1). Then, governments have adopted social distancing with the intention of diminishing the propagation rate of the disease and raising awareness of its citizens through new health, hygiene, behavioral habits and isolation (2).

According to Schiavi et al. (2020) (2), the COVID-19 pandemic represents a risk factor over individuals mental health. A stressing, traumatic, sudden and extremely unexpected event like the COVID-19 pandemic can cause post-traumatic stress disorder (PTSD) (3), which affects essential characteristics of sexual function, like the sensation of safety, self-efficacy and the capability of connecting with others (4).

The Impact of Event Scale Revised (IES-R) (5) questionnaire has been used to assess the PTSD triggered by COVID-19 pandemic (6). According to Letica-Crepulja et al. (2019) (7), PTSD can be used as a predictor parameter for sexual dysfunction. Because of that we hypothesize that COVID-19 pandemic can have a negative impact on sexual function.

Sexual function is a relevant component that contributes to individuals quality of life, and the negative correlation between psychological state and sexual function are well known (4), but little is known about the COVID-19 pandemic impact over sexual function in Latin American population, a region with peculiar sociocultural characteristics, not only because of its geographical proximity, which has cultural similarities (historical, linguistic, religious and political experiences) (8).

Considering that, the aim of our study was to investigate the interaction between the COVID-19 pandemic, demographic characteristics and erectile/sexual (E/S) function in Latin America.

## MATERIALS AND METHODS

### Study design, setting and participants

Cross-sectional study based on an anonymous web survey, through the Google Forms platform, provided in Portuguese and Spanish languages for the Latin American population (See supplementary Appendix-1).

The research was conducted from July to September 2020, proposed by the UroPhysiotherapy Laboratory researchers from the Post-graduate Program in Rehabilitation Science of the Federal University of Alfenas, after approval from the Institutional Review Board (IRB) of the university’s ethics and research committee (IRB number 34056120.7.0000.5142, Approval number 4128647), following the ethical precepts regulated by Resolution n. 466/12 of the National Health Council and the Helsinki Declaration requirements.

The research was released to the public with an invitation to fill the Google Forms survey through social media (WhatsApp, Facebook, Instagram), UNIFAL-MG communication websites, local newspapers, national and international symposia; reaching for individuals over 18 years old, sexually active, and available to fill the survey through a cell phone, computer or tablet. The Informed Consent Form was made available in the same Google Forms page.

The sample was composed by volunteers who answered the questions, recruited by convenience. The exclusion criteria were individuals under 18 years old, those that were not considered Latin Americans, as well as those who did not consent to the use of their data.

The research followed the Good Clinical Practice Guidelines, adopting the Strengthening the Reporting of Observational studies in Epidemiology (STROBE) guidelines.

### Measurement and quantitative variables

PTSD triggered by the COVID-19 pandemic: The isolation/social distancing measures during the COVID-19 pandemic were considered as triggering events to PTSD, which was investigated by the validated IES-R, asking the participants to consider the memories triggered by COVID-19 in the past seven days to answer the questionnaire. The IES-R is a self-applicable questionnaire originally developed in English language (5), translated and validated to Portuguese language by Santesso et al., (2012) (9) and to Spanish by Caamaño et al., (2011) (10). The scale is composed of 22 items distributed in three subscales (avoidance, intrusion and hyperarousal domains), each question varies from zero to four (0–4) points, total score ranging from 0 to 88, meaning that a higher score implies greater impairment. Cut-off point: 24 points, classified in: ≥ 24: PTSD is a clinical concern - higher score means a higher degree of PTSD; ≥ 33: best cut-off point for a likely PTSD diagnosis; ≥ 37: extreme PTSD, with enough consequences to cause immune system suppression, even 10 years after the triggering event.

Sexological outcomes: The sexual/erectile function was investigated considering the past four weeks, compared with before the COVID-19 pandemic, using the following variables:

Female sexual function: Clinical condition associated with the sexual act, it was investigated by the validated Female Sexual Function Index (FSFI) (11), self-applicable translated questionnaires for both Portuguese (12) and Spanish (13) languages. The FSFI questionnaire analyzes sexual response, considering desire, arousal, lubrication, orgasm, satisfaction and pain. Total score is calculated by adding the six scores weighted by the respective factor of each domain, varying from two (worst sexual function) to 36 (best sexual function); Cut-off point: 26.55, classified as: without sexual dysfunction: ≥ 26.55; with sexual dysfunction: <26.55 (11, 14).

Male erectile function: Male sexual function, a man’s clinical condition linked to the sexual act, was investigated by the Erectile Function domain (IIEF-5) (15) from the International Index of Erectile Function (IIEF) (16), with the purpose of measuring erectile function in a simple and direct way. IIEF was translated and validated in Portuguese language by Gonzales et al. 2013 (17) and Spanish by Zegarra et al. 2011 (18). IIEF-5 consists of five questions and the total score can vary from 5 to 25 points. A score lower than 22 is indicative of erectile dysfunction (15). Therefore, the following variables were considered: IIEF-5 total score: from five (05) (worst erectile function) to 25 (best erectile function); Cut-off point: 22, classified as: Without Erectile Dysfunction: ≥ 22; With Erectile Dysfunction: <22.

### Sex life aspects

Presence and frequency of sexual activity were investigated and classified as: present (increased frequency; no change in frequency; decreased frequency); suspended or no sexual activity; as well as sexual complaint (never displayed; previously presented; currently without complaint; currently present); partner at home during breakout of COVID-19 pandemic (lives with or without partner) and personal impression of the pandemic impact over sex life (numerical analog scale from 0 to 5).

Demographic data: Gender (male, female); age (18-33 years; 34-77 years, based on sample median); partner cohabitation status (living with or without partner); educational level (less than 10 years of education; 10 or more years of education) and family income (up to 2 minimum wages, 3 or more minimum wages).

### Bias

The study was performed anonymously, thus avoiding participants to be afraid or ashamed to answer questions of sexual nature. The researchers strived in divulgating the study within the Latin American population, encompassing most of Latin America countries and providing the questionnaire in Portuguese and Spanish.

### Statistical Analysis

The binary categorical variables (demographic and sexual dysfunction) were presented in absolute and relative frequencies, while the continuous variables (IES-R scores) were presented in central tendency values (average) and dispersion (standard deviation).

The cut-off point of 33 years old was the median of the sample, in order to create equivalent groups. The comparison for the total score average, pandemic impact and the three domains, between males and females, with and without indicative report for sexual dysfunction was performed by Student’s T test.

Bivariate logistic regression followed by a multivariate adjusted model (also adjusted for multicollinearity) were used. In all models, significance was analyzed by Wald test, considering p<0.05. All associations were evaluated by odds ratio (OR) values (confidence interval of 95%). The analysis were performed in the 4.0.0 version of the statistical software R (https://www.r-project.org/)

## RESULTS

**Figure 1:**
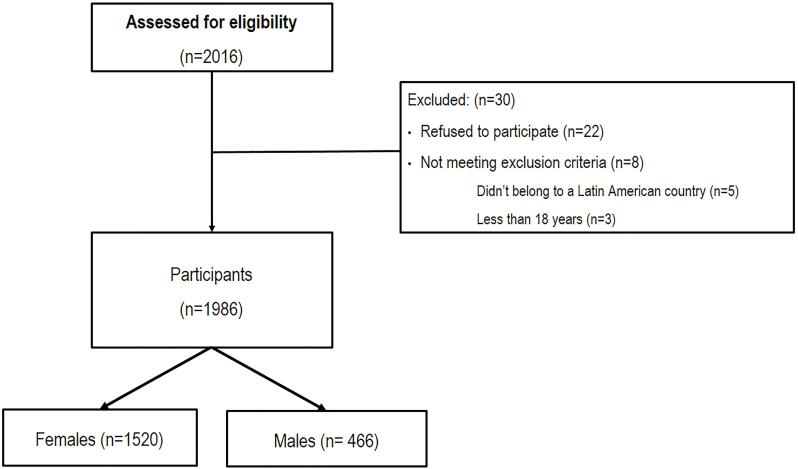
Flow diagram of the study.

As shown in Figure-1, this study’s questionnaires were answered by 2016 individuals, of whom 30 were excluded (22 refused to participate, five didn’t belong to a Latin American country and three had less than 18 years), remaining 1986 participants (466 males, 1520 females) from 17 Latin American countries (Brazil, Chile, Colombia, Argentina, México, Costa Rica, El Salvador, Bolivia, Ecuador, Perú, Venezuela, Nicaragua, Panamá, Guatemala, Paraguay, Puerto Rico, Uruguay).

The impact of the COVID-19 pandemic measured with IES-R was 36.7(±19.66), considering 33 the best cut-off point for PTSD diagnosis. It was found in the FSFI and IIEF-5 questionnaires that 37.5% of the participants had sexual disfunction. Also, the participants classified their impression of the pandemic impact on sex life, in a numeric scale from 0 to 5, which resulted in 2.45 (±1.78) (Table-1).

**Table 1:** Demographic characteristics, impact of event (IIES-R) and sexological outcomes during COVID-19 pandemic.

Variables		Participants
**Gender** f(%)		
	Male	466 (23.4)
	Female	1520 (76.6)
Age range* f(%)		
	18 - 33 years old	1050 (52.9)
	34 - 77 years old	933 (47.1)
**Partner cohabitation status f**(%)		
	Living with partner	1002 (50.4)
	Living without partner	984 (49.6)
**Educational level f(**%)		
	10 or more years of study	1636 (82.3)
	Less than 10 years of study	350 (17.7)
**Family income** f(%)		
	Three or more minimum wages	1475 (74.2)
	Until 2 minimum wages	511 (25.8)
Pandemic Impact** M(±SD)		
	Total score	36.37 (±19.66)
	Intrusion	12.61 (±7.86)
	Avoidance	13.48 (±7.53)
	Hyperarousal	10.27 (±6.25)
Sexual Function*** f(%)		
	Without sexual dysfunction	1242 (62.5)
	With sexual dysfunction	743 (37.5)
**Impression of the pandemic impact on sexual life** M(±SD)		2.45 (± 1.78)
**Sexual Activity** f(%)		
	Had not sexual activity and continued not to	124 (10.7)
	Decreased in frequency	426 (36.8)
	Suspended	142 (12.2)
	Without change	341 (29.2)
	Increased	122 (11.1)
**Sexual Complaint** f(%)		
	Never presented	533 (47.8)
	I had earlier, but currently I have no complaints	322 (27)
	I currently have a sexual complaint	280 (24.4)

The data are presented in absolute (f) and percent (%) frequencies as well as mean (M), standard deviation (SD).

* The cut-off point of 33 years old (median of the sample, in order to create equivalent groups.

** The Event Impact Scale - Revised (IES-R) questionnaire was used to investigate the COVID-19 pandemic impact, using the total score and the Intrusion, Avoidance, and Hyperarousal domains(9,10)

*** Sexual function was investigated using the Female Sexual Function Index (FSFI: ≤ 26.55) (14) and International Simplified Erectile Function Index (IIEF-5: ≤ 22) (15)

Comparing individuals with and without E/S dysfunction we found that individuals with E/S dysfunction had a higher IES-R score: total score (male [p<0.001]: without erectile dysfunction (ED): 26.01 [±19.25], with ED: 36.5 [±19.10]; female [p 0.001]: without sexual dysfunction (SD): 36.55 [±19.10], with SD: 41.28 [±18.99]); intrusion domain (male: p<0.001; female: p=0.011); avoidance domain (male: p<0.001; female: p=0.003) and hyperarousal domains (male: p<0.001; female: p<0.001) in both genders (Table-2).

**Table 2:** Relationship between the IES-R comparing individuals with and without erectile/sexual dysfunction. Analysis stratified by gender.

IES-R	Male (n=466)			Female (n=1520)		
	Without ED* (n=291)	With ED* (n=175)	p-value	Without SD* (n=952)	With SD* (n=568)	p-value
Total Score	26.01 (±19.25)	36.50 (±19.10)	<0.001	36.55 (±19.10)	41.28 (±18.99)	0.001
Intrusion	9.94 (±7.54)	13.88 (±7.13)	<0.001	13.74 (±7.53)	14.71 (±7.10)	0.011
Avoidance	8.88 (±7.46)	12.82 (±7.71)	<0.001	12.58 (±7.65)	14.50 (±7.80)	0.003
Hyperarousal	7.17 (±6.04)	9.80 (±5.98)	<0.001	10.23 (±6.03)	12.06 (±6.15)	<0.001

The Table shows the Impact of Event Scale Revised (IES-R) total score and the Intrusion, Avoidance and Hyperarousal domains (9, 10)

*** Sexual function was investigated using the Female Sexual Function Index (FSFI: ≤ 26.55) (14) and International Simplified Erectile Function Index (IIEF-5: ≤22) (15)

Test T Student (p=0.05)

ED = Erectile dysfunction; SD = Sexual dysfunction

In the association between demographic characteristics and E/S dysfunction evaluated by an unadjusted model we didn’t observe an association between gender (p<0.000 [CI 0.79-1.22]), however we did observe between the age group of 18-33 years old (p<0.000 [CI 0.69-1.00], OR 0.83), marital status of living without a partner (p<0.000 [CI 1.45-2.09], OR 1.74), family income of up to 2 minimum wages (p<0.011 [CI 1.05-1.59], OR 1.30) and the E/S function. On the other hand in the adjusted model, only marital status maintained the association (p<0.000 [CI 1.42-2.13]), as those who do not live with their partner are 74% more likely to have E/S dysfunction (OR 1.74).

In the association between the IES-R and IIEF-5 or FSFI, we found a positive association in the IES-R total score (p<0.00 [CI 1.01-1.02], OR 1.02), intrusion domain (p<0.00 [CI 1.01-1.04], OR 1.02), avoidance domain (p<0.00 [CI 1.02-1.05], OR 1.03) and hyperarousal domain (p<0.00 [CI 1.03-1.06], OR=1.05). It is observed that with each score taken from the IES-R questionnaire, the chance of sexual dysfunction increases (OR>1) or decreases (OR<1).

In the interaction model between IES-R and the significant demographic variable (marital status) for the E/S function, we found that those who live with a partner had greater impact of the pandemic on E/S function in the total score and the avoidance and hyperstimulation domains, but not in the intrusion domain (Table-3).

**Table 3:** Interaction between the Impact of Event Scale Revised (IES-R) and the significant demographic characteristics for the erectile/sexual function.

Variables		No interaction OR (CI 95%)	Principal effects OR (CI 95%)	Interaction term OR (CI 95%)
**IES-R total score**				
	Marital status	1.62	2.70	0.98
	Without partner	(1.34 – 1.95)	(1.80 – 4.07)	(0.97 – 0.99)
	IES-R total score	1.02 (1.01 – 1.02)	1.02 (1.01 – 1.03)	---
**Intrusion domain**				
	Marital status	1.68 (1.39 – 2.02)	2.30 (1.56 – 3.41)	0.97 (0.95 – 1.01)
**Without partner**				
	Intrusion domain	1.02 (1.01 – 1.03)	1.03 (1.02 – 1.05)	---
**Avoiding domain**				
	Marital status	1.63	2.43	0.97
	Without partner	(1.35 – 1.97)	(1.69 –3.50)	(0.94 – 0.99)
	Avoiding domain	1.03 (1.02 – 1.04)	1.05 (1.03 – 1.07)	---
**Hyperarousal domain**				
	Marital status	1.59 (1.32 – 1.92)	2.79 (1.92 – 4.07)	0.94 (0.92 –0.97)
**Without partner**				
	Hyperarousal domain	1.04 (1.03 – 1.06)	1.07 (1.05 – 1.10)	---

Verified by a Multivariate Logistic Regression model

**IES-R** = Impact of Event Scale – Revised; **OD** = odds ratio; **CI** = Confidence Interval

## DISCUSSION

This study demonstrated the relation between the COVID-19 pandemic and PTSD, with a negative interaction between IES-R and erectile/sexual function on the Latin American population. PTSD was a predictor of sexual dysfunction like in the Letica-Crepulja 2019 study (7). During the COVID-19 pandemic, Fang et al. (2020) also used the IES-R and IIEF-5 questionnaires to evaluate male healthcare professionals. Their findings corroborate with our study by the negative interaction found between them (6).

In addition, among the demographic factors, the participants marital status stood out, demonstrating that individuals who live without partner presented higher prevalence of erectile/sexual dysfunction; while individuals who are living with partner presented higher pandemic impact over erectile/sexual function. We hypothesize that during the COVID-19 pandemic people who live without a partner have greater difficulties in engaging in sexual intercourse, but those who live with their partners may have more impact because they have to stay together at all times, affecting their relationship and, consequently, their sexual lives. Additionally, it can be harder to engage in moments of sexual activity with their families staying at home all day long.

Schiavi et al. (2020) in their study with females during the COVID-19 pandemic found a lower total FSFI score among women with higher level of education, but in this study no relation with educational level was identified (2).

During the pandemic, Mollaioli et al. interviewed 2,608 sexually active individuals, and they found a prevalence of 18.5% for erectile dysfunction in males and 28.8% for sexual dysfunction in females (19). In our study, it was found in 37.55% of males and 37.37% of females. There was a bigger participation of females, but no differences were found in the E/S function (p<0.000 [CI 0.79-1.22]).

This study did not find any relation between age and the presence of sexual dysfunction, which differs from the studies carried out before the COVID-19 pandemic, which found a strong influence of age over erectile dysfunction (20).

FSFI was also used in Schiavi et al. and Yuksel. et al. studies in the COVID-19 pandemic in females. Both observed worse scores compared to data prior to the COVID-19 pandemic (2, 21).

Pennanen-Iire et al. (2020) reported that the stress triggered by the COVID-19 pandemic for long cohabiting times could compromise the couple’s sex life, including an increase in anxiety and fear of failing in sexual performance (22). Associated with this, we must consider the limitation of individual space and the difficulty to find moments of intimacy while the family stays at home during the whole time (23).

To our knowledge, this is the first study about sexual function during the COVID-19 pandemic in Latin America. The study was performed online, which facilitated the access to individuals from 17 out of the 20 Latin American countries, allowing for reflection about the reality experienced by the population during the COVID-19 pandemic.

This study compared the male and female population using specific instruments to each population and considering aspects related to the male and female sexual function, such as penetrative vaginal sex and erection, respectively; which the researchers considered a limitation of the used instruments.

Therefore, we emphasize the importance of developing, in future studies, questionnaires that are more inclusive in relation to non-penetrative sex, masturbation and non-heterosexual orientations. In the same way, to date there are no validated questionnaires for the evaluation of general sexual function in males.

Clinical guidelines during the COVID-19 pandemic are being consolidated for Latin America professionals (24) and we believe that this study can have clinical implications that contribute to the knowledge about the COVID-19 pandemic impact over erectile/sexual function of Latin Americans, allowing for future intervention proposals that consider sexual health care in post-pandemic times. A study by Gomes et al. 2021 shows that more quality research and apps are necessary before the widespread use of mobile health technologies (25).

The COVID-19 pandemic and its implications, such as quarantine, labor or wage losses, close familiar interaction with all inhabitants on the domestic ambient, privation of liberties both at home and on the outside, privation of routine activities, fear of the unknown and the repercussions of the disease, limitation of the routine consultations for physicians and other health professionals, double workday for some and idleness for others, among many other aspects may have contributed to the impact on sexual function, drawing a necessary and special attention for the years to come.

More attention is needed for the Latin American population, especially non-heterosexual individuals. Future studies should seek for alternatives in remote solutions and treatments for people whose sexual function was affected by the COVID-19 pandemic and apply it in these times, and after the pandemic ends.

Our study presents limitations that are inherent to online surveys, such as containing information that is not completely understood by the respondents, demanding for internet access and also proficiency in technological resources. Moreover, the propagation of the survey by the researchers’ and collaborators’ social media may have been biased, since members of other social media may have not been reached. Similarly, since the invitations are open to contact networks, normally those who are more interested and participative tend to answer readily. On the other side, the access through social media can favor larger sample size for online surveys.

It is important to consider that the individual’s state prior to the pandemic was not consulted. Furthermore, quarantine conditions may have differed among countries, which can influence the interpretation of results.

## CONCLUSIONS

An interaction between the COVID-19 pandemic impact and erectile/sexual function was found. Individuals that do not live with their partners presented higher prevalence of sexual dysfunction. However, the pandemic triggered greater impact over the erectile/sexual function of people who live with a partner.

## ABBREVIATIONS

COVID-19: Coronavirus disease 2019
E/S: Erectile/sexual
IIEF-5: Simplified International Index of Erectile Function
FSFI: Female Sexual Function Index
PTSD: Post-traumatic stress disorder
IES-R: Impact of Event Scale Revised
